# The CouPSTU and TarPQM Transporters in *Rhodopseudomonas palustris*: Redundant, Promiscuous Uptake Systems for Lignin-Derived Aromatic Substrates

**DOI:** 10.1371/journal.pone.0059844

**Published:** 2013-03-28

**Authors:** Robert C. Salmon, Matthew J. Cliff, John B. Rafferty, David J. Kelly

**Affiliations:** 1 Department of Molecular Biology and Biotechnology, The University of Sheffield, Sheffield, United Kingdom; 2 Manchester Institute of Biotechnology, The University of Manchester, Manchester, United Kingdom; University of South Florida College of Medicine, United States of America

## Abstract

The biodegradation of lignin, one of the most abundant carbon compounds on Earth, has important biotechnological applications in the derivation of useful products from lignocellulosic wastes. The purple photosynthetic bacterium *Rhodopseudomonas palustris* is able to grow photoheterotrophically under anaerobic conditions on a range of phenylpropeneoid lignin monomers, including coumarate, ferulate, caffeate, and cinnamate. RPA1789 (CouP) is the periplasmic binding-protein component of an ABC system (CouPSTU; RPA1789, RPA1791–1793), which has previously been implicated in the active transport of this class of aromatic substrate. Here, we show using both intrinsic tryptophan fluorescence and isothermal titration calorimetry that CouP binds a range of phenylpropeneoid ligands with *K*_d_ values in the nanomolar range. The crystal structure of CouP with ferulate as the bound ligand shows H-bond interactions between the 4-OH group of the aromatic ring with His309 and Gln305. H-bonds are also made between the carboxyl group on the ferulate side chain and Arg197, Ser222, and Thr102. An additional transport system (TarPQM; RPA1782–1784), a member of the tripartite ATP-independent periplasmic (TRAP) transporter family, is encoded at the same locus as *rpa1789* and several other genes involved in coumarate metabolism. We show that the periplasmic binding-protein of this system (TarP; RPA1782) also binds coumarate, ferulate, caffeate, and cinnamate with nanomolar *K*_d_ values. Thus, we conclude that *R. palustris* uses two redundant but energetically distinct primary and secondary transporters that both employ high-affinity periplasmic binding-proteins to maximise the uptake of lignin-derived aromatic substrates from the environment. Our data provide a detailed thermodynamic and structural basis for understanding the interaction of lignin-derived aromatic substrates with proteins and will be of use in the further exploitation of the flexible metabolism of *R. palustris* for anaerobic aromatic biotransformations.

## Introduction

**Figure 1:**
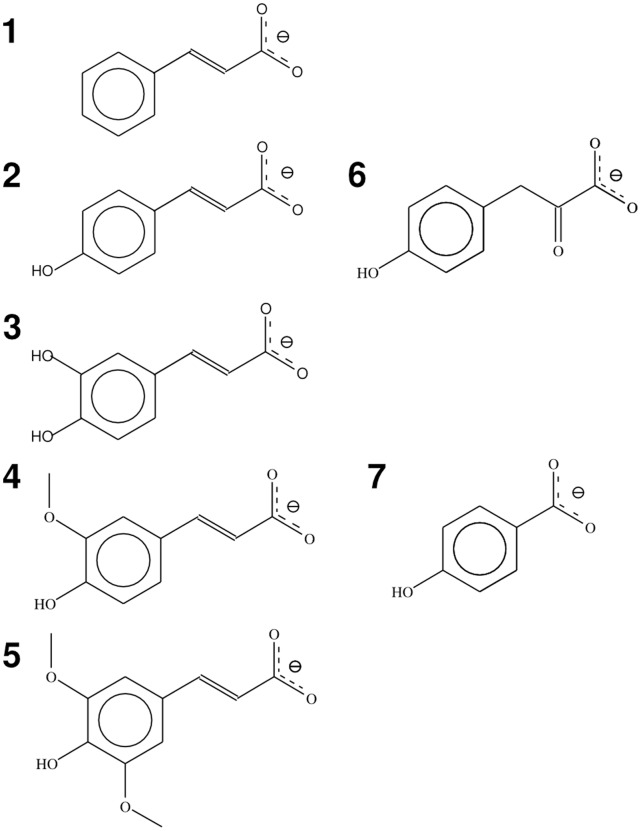
Structures of lignin derived aromatic monomers and related compounds used in this study. Key: 1; cinnamate, 2; *p*-coumarate, 3; caffeate; 4; ferulate, 5; sinapate, 6; 4-hydroxyphenylpyruvate, 7; 4-hydroxybenzoate.

Almost one third of the world’s dry plant mass is made up of the complex compound lignin, which is formed by the polymerisation of a wide range of aromatic phenylpropeneoid monomers [1]. In the environment, the biodegradation of lignin occurs through a mixed population of microorganisms that co-operate to break down the individual constituents at the various stages of degradation. A population of bacteria and white-rot fungi such as *Phanerochaete chrysosporium* secrete a combination of laccases and peroxidases that help to cleave the majority of the more stable bonds, particularly the β-aryl ether linkages that are a key part of the polymeric structure [2]. This results in a mixture of aromatic monomers that are more accessible for degradation [3]. Among the most numerous of these aromatic monomers are a range of structurally related cinnamic acids [4], including cinnamate itself ((*E*)-3-phenyl-2-propenoic acid), *p-*coumarate ((*E*)-3-(4-hydroxyphenyl)-2-propenoic acid), caffeate ((*E*)-3-(3,4-dihydroxyphenyl)-2-propenoic acid) and ferulate ((*E*)-3-(4-hydroxy-3-methoxyphenyl)-2-propenoic acid). The structures of these compounds are shown in Fig. 1.

*Rhodopseudomonas palustris* is a purple non-sulphur Gram-negative photosynthetic bacterium that is found in a wide variety of environments and which has an extremely complex and flexible metabolism, as highlighted by the genome sequence of the best studied strain, CGA009 [5]. It can degrade a wide variety of aromatic compounds under both aerobic and anaerobic conditions [6] and has become a model organism for the study of aromatic catabolism under anaerobic conditions. It has long been established that anaerobic breakdown of such compounds by *R. palustris* is carried out through the central intermediate benzoyl-CoA [7], [8] and a downstream ring cleavage pathway [9]. In more recent studies, a range of lignin-derived phenylpropenoic acids have been shown to be degraded anaerobically by *R. palustris* via initial conversion to a Coenzyme A (CoA) derivative followed by metabolism to benzoyl-CoA and the subsequent ring cleavage pathway [10].

Initial studies into the peripheral pathways that degrade these phenylpropeneoid monomers began with the proposition of two possible routes of degradation derived from studies into ferulate degradation carried out in other organisms such as *Pseudomonas acidovorans* [11] and *Delfitia acidovorans* [12]; the non-β-oxidation and β-oxidation pathways [13]. In the non β-oxidation pathway the CoA derivative is first converted to an aldehyde with the loss of acetyl-CoA, thus shortening the side chain by a C2 unit. The aldehyde is then oxidised to a carboxylic acid, which is derivatized with CoA once more before conversion to benzoyl-CoA. In contrast, in the β-oxidation pathway the CoA remains attached to the intermediates, which undergo chain shortening by hydration and oxidation with a C2 unit removed as acetyl-CoA in the last step before conversion to benzoyl-CoA via an acyl-transferase. A β-oxidation pathway was originally proposed in *R. palustris* for the side-chain degradation of saturated phenylalkane carboxylic acids [14].

To investigate which of these two mechanisms was most likely to be involved in coumarate degradation in *R. palustris*, Pan *et al*. [13] carried out a transcriptomic and proteomic study on coumarate or succinate supplemented *R. palustris* cells growing in steady-state chemostat culture. This revealed that a cluster of genes encoding candidate enzymes of the non β-oxidation pathway were highly up-regulated in the presence of coumarate [13], suggesting that this was likely to be the major pathway used for coumarate degradation. The *p*-coumarate CoA ligase (CouB) and enoyl-CoA hydratase (CouA) encoded in this cluster were then biochemically characterised in a later study [10] and shown to be regulated in expression by CouR (RPA1794), a MarR type regulator that responds specifically to *p*-coumaroyl CoA rather than *p*-coumarate itself.

Other genes in the non β-oxidation pathway cluster suggest two distinct types of aromatic transport systems might be involved in growth on coumarate and related compounds. These genes encode an ABC-type uptake system (*rpa1789* and *rpa1791-1793*) and a tripartite ATP-independent periplasmic (TRAP) transporter [15] encoded by *rpa1782-1784*. It has recently been shown that the genes encoding these specific TRAP and ABC systems are up-regulated in the presence of *p*-coumarate under the control of the CouR regulator [10], [16]. In a study comparing thermal denaturation profiles in the absence and presence of potential ligands, the purified periplasmic binding-protein RPA1789 from the ABC system was initially suggested to bind several cinnamic acid derivatives [17]. A further investigation [18] characterised the binding of coumarate to RPA1789 by isothermal titration calorimetry (ITC) but reported a *K*_d_ value of 8.6 µM for this ligand, which is much higher than would be expected for a typical periplasmic binding-protein of an ABC transporter. Pietri *et al*. [18] also reported some initial structural studies of RPA1789 using X-ray scattering data, which showed the protein to have an overall shape similar to other periplasmic binding-proteins.

In this paper we compare the ligand specificity and binding affinities of the periplasmic binding proteins RPA1782 and RPA1789 from the TRAP and ABC systems of the non β- oxidation pathway gene cluster respectively. Data from a combination of fluorescence spectroscopy and ITC clearly show that both proteins have binding affinities in the nanomolar range for coumarate, caffeate and ferulate, with weaker (but still high affinity) binding of cinnamate. We also report a structural study of RPA1789. An initial structure of RPA1789 we had obtained after crystallisation in the absence of any added ligands had revealed the presence of 4-hydroxyphenylpyruvate (4-HPP) (a tyrosine metabolic intermediate) in the binding cleft of the protein, and we subsequently confirmed by fluorescence titration that the protein binds 4-HPP with high-affinity. After a denaturation and refolding protocol that removed this endogenous ligand contaminant, a 2.5 Å structure with coumarate bound and a 1.9 Å structure with ferulate bound were determined. In view of the functions of the cognate transporters, we propose the designations CouPSTU (**Cou**marate transport by RPA1789 and RPA1791-RPA1793) and TarPQM (**T**RAP transporter for **ar**omatic compounds; RPA1782-RPA1784) for the components of the ABC and TRAP transporters respectively.

## Materials and Methods

### Construction of Overexpression Plasmids

The *rpa1789* gene was amplified from *R. palustris* CGA009 genomic DNA via PCR using primers *rpa1789_F* (5′-ATTAACTACATATGGAAACTAACGAAATCACCATC-3′ *Nde*I site underlined) and *rpa1789_R* (5′-ATATTTTAGCGGCCGCCTTCACCATCACGTATTT -3′ *Not*I site underlined), which excluded the N-terminal signal sequence (protein residues 1–26). Amplified DNA was then cloned into the pET21a (+) vector via *Nde*I and *Not*I restriction sites to add a C-terminal His_6x_-tag to the recombinant protein. The construct pET1789 was transformed into *E. coli* DH5α and then subsequently into *E. coli* BL21 (DE3) for overproduction of protein. The *rpa1782* gene was amplified from *R. palustris* CGA009 genomic DNA via PCR using primers *rpa1782_F* (5′-ATTGTACTCGAGACAGGACAAAACTGTCAACTGG-3′, *Xho*I site underlined) and *rpa1789_R* (5′-ATCGAATTCTTACAGCCCCGCGTCGTACTT-3′, *Eco*RI site underlined), which excluded the N-terminal residues 1–19 of the protein signal sequence. Amplified DNA was then cloned into the pBAD/HisB vector via *Xho*I and *Eco*RI restriction sites to add an N-terminal His_6x_-tag to the recombinant protein. The construct pBAD1782 was then transformed into the *E. coli* TOP10 expression strain for protein overproduction.

### Overproduction and Purification of RPA1789

**Figure 2:**
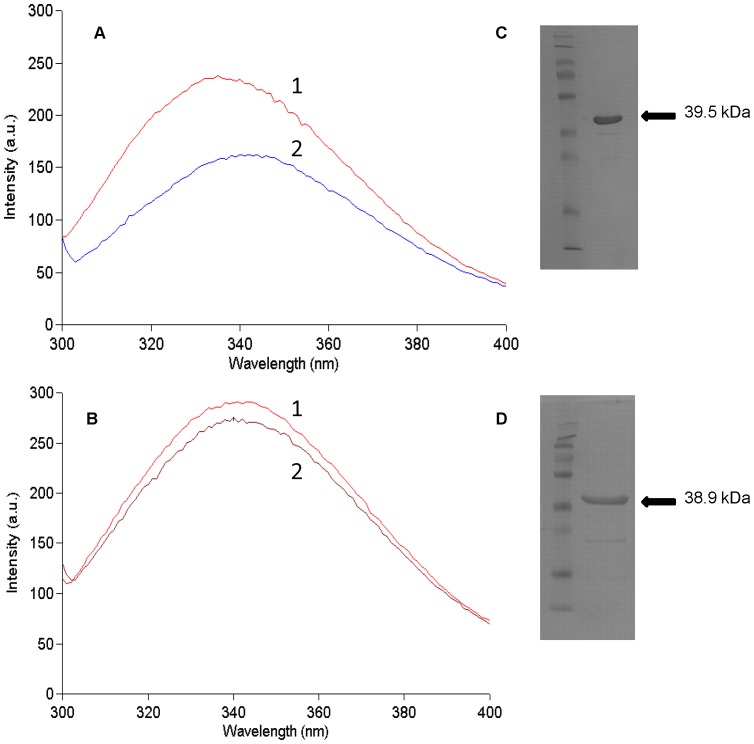
Changes in intrinsic tryptophan fluorescence of (A) CouP (RPA1789) and (B) TarP (RPA1782) upon addition of equimolar *p*-coumarate. Purified protein (0.2 µM final concentration in each case) in Tris-HCl buffer pH 7.4 was excited at 280 nm and the emission spectrum obtained (traces labelled 1). After addition of equimolar p-coumarate, the emission spectrum was obtained again (traces labelled 2). In (A) the coumarate induced quench was ∼ 30% while in (B) it was ∼ 5%. In (C) and (D) are shown Coomassie blue stained gels of the purified CouP (6 µg protein) and TarP (2.5 µg protein) respectively.

The *rpa1789* gene was over-expressed under the control of the isopropyl-β-D-thiogalactopyranoside (IPTG)-inducible T7 promoter contained in the pET1789 vector. *E. coli* BL21 (DE3) (pET1789) was grown to an OD_600 nm_ of 0.6 in LB medium containing carbenicillin (50 µg/ml) (Melford Laboratories, UK) at 37°C. Then, 0.4 mM IPTG was added and cells were incubated at 37°C with shaking at 250 rpm for a further 5 hours before being harvested by centrifugation (10,000×*g*, 10 mins, 4°C). Pelleted cells were resuspended in 20 mM sodium phosphate buffer pH 7.4 and broken by sonication (MSE soniprep; 4×20 s bursts). Soluble protein was then isolated by centrifugation (15,000×*g*, 25 min 4°C) to produce a cell-free extract (CFE). The CFE was loaded onto a Hitrap-HP Nickel affinity column (GE healthcare, UK) in binding buffer (20 mM sodium phosphate buffer pH 7.4, 500 mM sodium chloride, 20 mM imidazole). Bound protein was then eluted in a pure form (see Fig. 2) from the column by an imidazole gradient using elution buffer (20 mM sodium phosphate buffer pH 7.4, 500 mM sodium chloride, 500 mM imidazole).

### Overproduction and Purification of RPA1782

RPA1782 was overproduced in *E. coli* TOP10 (pBAD1782) cells. After growth at 37°C to an OD_600 nm_ of 0.6, cells were induced by addition of 0.002% (w/v) arabinose and shaken at 250 rpm for 3 hours before harvesting by centrifugation (10,000×*g*, 10 mins, 4°C). Cell-free extracts were prepared as above. Initial protein purification by His-trap affinity chromatography resulted in a ∼57 kDa protein predominating over the ∼38 kDa RPA1782. This contaminant was N-terminally sequenced and identified as the *E. coli* chaperone protein GroEL. In order to remove contaminating GroEL, CFE was bound to a His-trap column and washed with 6 M urea, which eluted GroEL from the column; recombinant RPA1782 remained bound due to its N-terminal His_ 6x_-tag. RPA1782 was refolded on the column by washing with 75 ml 20 mM sodium phosphate buffer pH 7.4 and was then eluted via a 0–0.5 M imidazole gradient.

### Unfolding and Refolding of RPA1782 and RPA1789

Pure protein was unfolded by dialysis against 1 L of 6 M urea for 16 hours at 4°C. Refolding was effected by dialysis against 2 L of refolding buffer (50 mM Tris-HCl pH 7.2 plus 100 mM NaCl) for 8 hours, the buffer being changed every 2 hours with a final overnight dialysis. Once removed from the dialysis tubing, protein was centrifuged (13,000×g, 10 mins, 4°C) to remove any precipitated misfolded protein.

### Fluorescence Spectroscopy

Changes in the UV fluorescence of intrinsic tryptophan residues in RPA1782 and RPA1789 were measured using a Cary Eclipse fluorimeter (Varian Ltd, UK) in 10 mM Tris-HCl buffer pH 7.4 at 30°C in a 3 ml volume. Excitation of samples was at 280 nm (5 nm slit width) and emission was recorded at 300–400 nm (20 nm slit width). Ligand titrations were performed with 0.2 µM protein with λ_ex_ 280 nm λ_em_ 340 nm using 5 nm excitation and 20 nm emission slit widths respectively. Except where indicated, data from three independent titrations was used to calculate *K*_d_ values, by fitting to the quadratic equation for tight binding as previously described [19].

### Isothermal Titration Calorimetry (ITC) with RPA1789

ITC experiments were carried out using a VP-ITC calorimeter (Microcal, Inc., UK). Ligand stock solutions for ITC experiments were made up in aliquots of the final refolding buffer that RPA1789 was dialysed against (50 mM Tris-HCl pH 7.2 plus 100 mM NaCl) and all reactions used the same buffer. The reaction cell contained 1.8 ml of 50 µM protein in refolding buffer. Ligand titrations were carried out at 20°C (except caffeate which was carried out at 25°C) by the injection of 20×15 µl aliquots of 400 µM ligand, from a 300 µl syringe, for a duration of 35 seconds at 7 minute intervals. After taking into account the baseline and any heats of dilution by the ligand, all titrations were integrated using the ORIGIN software programme associated with the calorimeter then exported to in-house numerical routines for data fitting to the Weissman isotherm.

### Crystallisation of RPA1789

For crystallization, the purified RPA1789 was concentrated to 6 mg ml^−1^ in 10 mM Tris-HCl buffer, pH 7.4 and tested for crystallization with a variety of commercial screens. Subsequent optimization resulted in the growth of X-ray diffracting crystals in a condition comprising 0.1 M sodium acetate, pH 4.5, 25% ^w^/_v_ PEG3350. The crystals formed with a thin needle-like morphology at 17 °C overnight. Ligand containing crystals were obtained in the same manner, with ligand (coumarate or ferulate) being added to the protein sample at 5 mM prior to crystallisation.

### Data Collection and Structure Determination

Data were collected from all crystals at the Diamond Light Source (near Oxford, UK) on beamline station I02 having been flash-cooled in a N_2_ gas stream at 100 K with prior cryo-protection via the addition of 15% ^v^/_v_ glycerol to the mother liquor. The data were processed with XDS and merged using Xscale [20] before molecular replacement was carried out using PHASER [21] as implemented in the Collaborative Computational Project, Number 4 (CCP4) software suite [22] with a model constructed using the Phyre^2^ server [23] based upon PDB entry 3UK0 for *R. palustris* RPD1889 extracellular binding receptor protein. The resultant maps were examined using COOT [24] and adjustments made to the models before refinement with REFMAC [25]. Solvent molecules were added to all the structures in the latter stages of refinement and ligand coordinate and description files for use in refinement created using JLIGAND. The structures were validated using COOT [24] and MOLPROBITY [26]. The final models are complete for each form of the protein apart from residues 27 in the ferulate complex, the latter residues of the C-terminal hexahistidine tags and the signal sequence (residues 1–26), which had been deleted from the expression construct. Structure factors and coordinates have been deposited at the PDB with the accession codes 4JB2 (ligand free) and 4JB0 (ferulate bound). Structure figures were generated using Pymol or Ligplot^+^ [27].

## Results

### Tight Binding of Lignin-derived Aromatic Compounds to RPA1789 Revealed by Fluorescence Spectroscopy

To characterise the binding of lignin-derived aromatic compounds to RPA1789, the intrinsic fluorescence of tryptophan residues was monitored to measure ligand induced conformational changes, as used in our previous work [19], [28]. Excitation of unfolded, refolded and extensively dialysed RPA1789 at 280 nm resulted in an emission maximum at 335 nm. Addition of 0.2 µM of coumarate, ferulate, cinnamate or caffeate to 0.2 µM of RPA1789 resulted in a large (∼25–50%) quench for each ligand and a shift in the emission maximum to ∼340 nm; Fig. 2a shows typical changes upon coumarate binding. 4-hydroxybenzoate did not produce any fluorescence change with RPA1789, indicating that possession of the propanoid side chain is essential for ligand-binding to this protein (see Fig. 1). The potential ligands 3,4-dihydroxyhydrocinnamate (hydrocaffeate), 4-methoxycinnamate and vanillate were so autofluorescent that no firm conclusions about binding could be made. However sinapate (3-(4-hydroxy-3,5-dimethoxyphenyl)-2-propenoic acid) produced a ∼ 5% quench indicating it might bind to the protein, but this ligand auto-oxidised easily in solution to a coloured product and titrations to obtain a *K*_d_ were unreliable.

**Figure 3:**
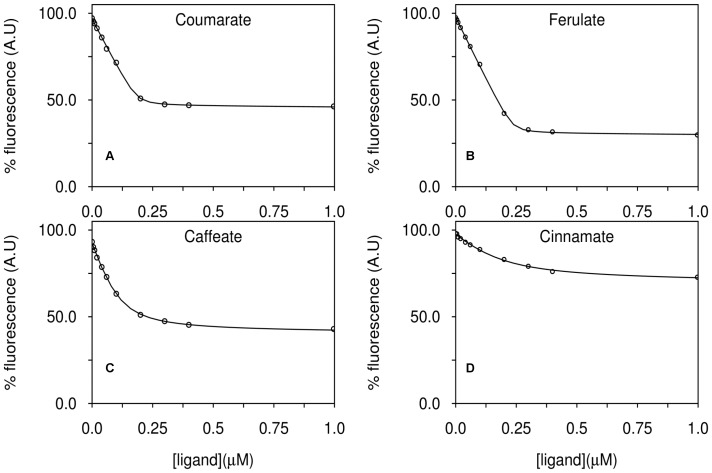
Fluorescence titrations of CouP (RPA1789) with lignin-derived aromatic ligands. The fluorescence emission of 0.2 µM CouP was followed at 340 nm during titrations with the indicated compounds; (A) coumarate, (B) ferulate (C) caffeate (D) cinnamate. In each case, data were fit to a single-site binding model and dissociation curves were plotted. The figure shows an individual representative titration.

Titrations of RPA1789 with those aromatic ligands producing a consistent fluorescence change are shown in Fig. 3. In each case a binding stoichiometry of approximately 1∶1 is apparent. For coumarate, caffeate and ferulate the shape of the plot indicates tight binding with calculated *K*_d_ values of 2.6 nM, 2.4 nM and 15 nM respectively (Table 1). The titration with cinnamate, which has no additional functional groups on the aromatic ring, showed a smaller total fluorescence change and a much higher calculated *K*_d_ of 88 nM (Table 1).

**Table 1:** Comparison of *K*_d_ values determined from fluorescence titrations with CouP and TarP.

	*K*_d_ (nM)	
Ligand	CouP (RPA1789)	TarP (RPA1782)
Coumarate	2.6**±**0.09	8±5
Caffeate	2.4**±**0.8	14±7
Ferulate	15**±**6	15±3
Cinnamate	88**±**8	50, 33

The values shown are averages plus errors from three independent titrations. The fluorescence changes during cinnamate titrations with TarP were highly variable, so the *K*_d_ values from two independent titrations are shown.

### Thermodyamic Analysis of Ligand Binding to RPA1789 by Isothermal Titration Calorimetry

**Figure 4:**
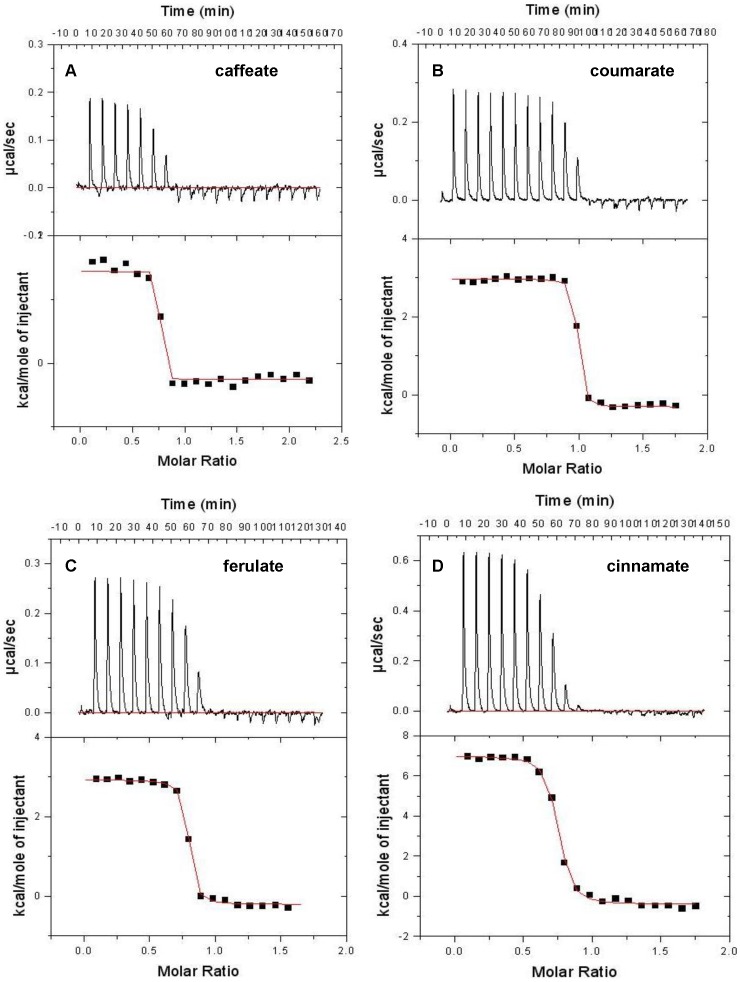
Isothermal titration calorimetry of ligand binding to CouP (RPA1789). Purified CouP (50 µM final concentration in dialysate reaction buffer) was titrated with (A) caffeate (B) coumarate (C) ferulate (D) cinnamate by automated ligand injections into the ITC reaction cell as described in Materials and Methods. The thermograms are shown in the top panel of each ligand dataset and the corresponding binding isotherms are shown in the lower panels. The solid lines in the lower panels are the fits to the data points, which were calculated using the ORIGIN software associated with the calorimeter. The resulting thermodynamic parameters are listed in Table 2. Molar ratio refers to moles of injectant per mole of protein.

Fluorescence spectroscopy highlighted four structurally related aromatic ligands that have sub-micromolar binding constants with RPA1789. The interactions of these ligands were also investigated by ITC methods. As well as giving additional information on the thermodynamics of the binding interactions, as ITC is a non-optical method it was particularly useful for aromatic ligands given their potential for inner filter effects and background emission artefacts in fluorescence studies. ITC titrations were carried out at 20°C (apart from caffeate which had a negligible enthalpy of binding at 20°C) and for each ligand it was apparent that endothermic heat changes occurred as ligand was bound (Fig. 4). This is expected of hydrophobic interactions where the entropy change associated with the displacement of water molecules from the binding site is the dominant favourable effect. The thermodynamic data are presented in Table 2. The *K*_d_ values measured by ITC are in general slightly weaker than those measured by fluorescence, but report a very similar range. Furthermore, the order of preference for the ligands detected by the two techniques is the same.

**Table 2:** Thermodynamic data derived from ITC of CouP (RPA1789) with the four lignin-derived aromatic ligands.

Ligand	Temp (°C)	ΔH(kcal mol^−1^)	ΔHdil(kcal mol^−1^)	n	ΔS(cal mol^−1^ K^−1^)	*K*_d_ (nM)
Coumarate	20	3.23±0.03	−0.26	0.993**±**0.003	46	17±0.06
Caffeate	25	1.78±0.05	−0.28	0.776**±**0.005	40	27±0.07
Cinnamate	20	7.45±0.08	−0.43	0.757**±**0.003	56	220±0.46
Ferulate	20	3.18±0.03	−0.24	0.803**±**0.002	43	84±0.02

ΔH is the binding enthalpy, ΔHdil refers to the heats of dilution of the ligands, n is the ligand binding stoichiometry and ΔS is the binding entropy.

In view of the tight binding of coumarate to RPA1789 evidenced by both ITC and fluorescence spectroscopy, the genome context of the *rpa1789* and *rpa1791-1793* transporter genes, their clustering with coumarate catabolic genes and their common regulation by CouR [10], [16], we propose that the components of this ABC transporter be designated CouP (RPA1789), CouS (RPA1791), CouT (RPA1792) and CouU (RPA1793).

### The Crystal Structure of Ferulate Bound CouP

**Figure 5:**
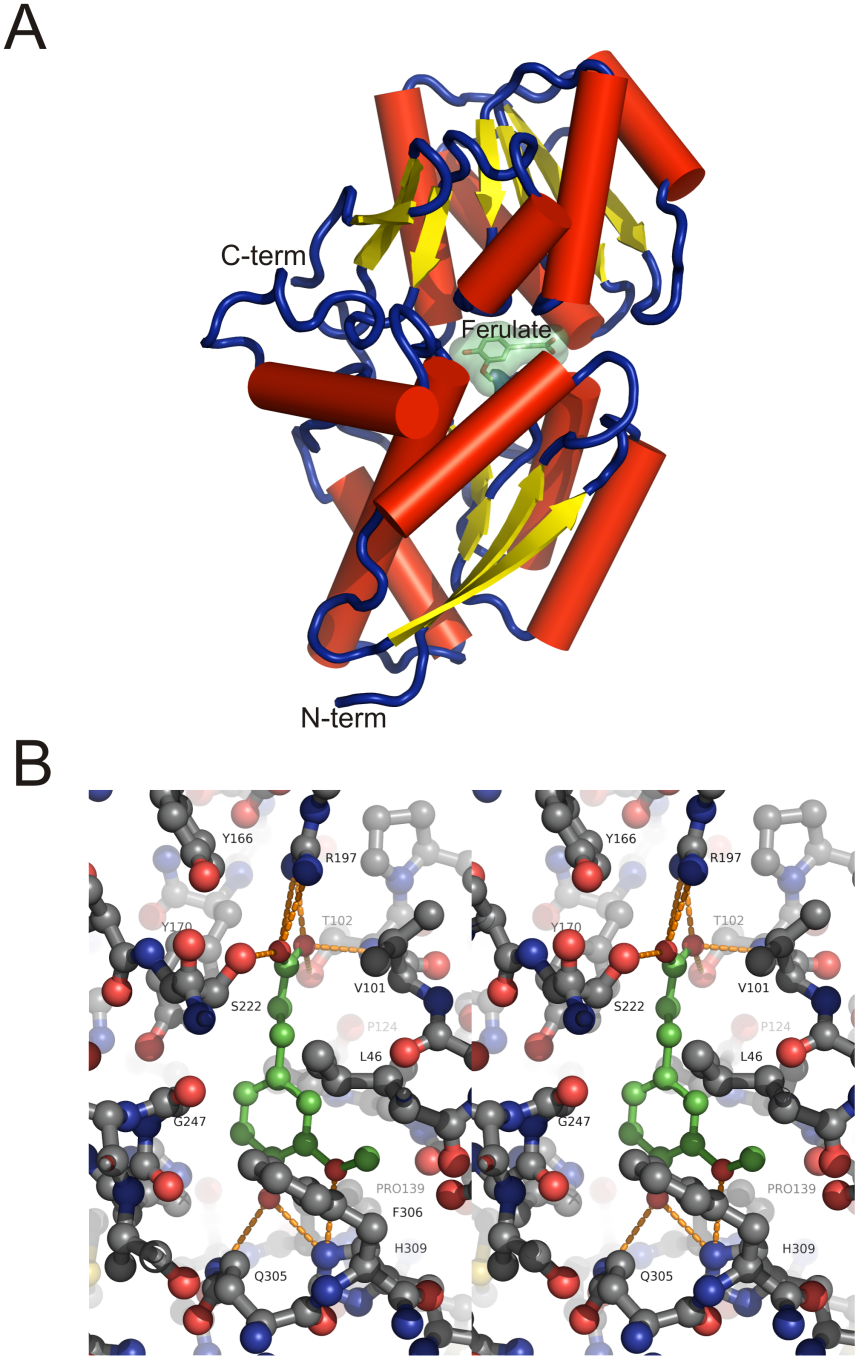
The 1.9Å resolution crystal structure of CouP (RPA1789) with bound ferulate. (A) Representation of the overall fold with ferulate positioned in the binding cleft. Alpha helices are represented by red cylinders, loops by blue strands and beta-sheets by yellow arrows. **(**B) Ferulate binding site. A stereo image is shown of ferulate (green carbon atoms & bonds) in the substrate binding pocket of CouP (dark grey carbon atoms & bonds). The end of the pocket proximal to the exterior solvent is uppermost and closed off by the sidechain of R197. Hydrogen bonds between the ferulate and protein are shown as dashed orange lines. The molecules are shown in ball-and-stick representation coloured by atom type.

Crystallographic studies were undertaken to elucidate the mechanism of ligand binding by CouP. An initial 1.5 Å resolution structure was obtained with protein that was not subjected to unfolding and refolding. Electron density that fitted 4-hydroxyphenylpyruvate (4-HPP; see Fig. 1) was found in the binding-cleft (data not shown) despite no ligand being added to the crystallisation reaction. Fluorescence titrations with 4-HPP confirmed that refolded CouP can bind this ligand with a determined *K*_d_ of 15±3 nM. The importance of removing endogenously bound ligand was shown in experiments with CouP that was not urea treated, where we found no evidence for cinnamate binding, and *K*_d_ values for coumarate, caffeate and ferulate that were 200-1,000-fold higher (by fluorescence titrations) than with refolded protein. Crystallisation experiments were repeated using protein that was subjected to extensive unfolding and refolding dialysis as described in Materials and Methods and the X-ray diffraction data revealed a structure with only solvent molecules in the expected binding pocket. Crystal trials and data collection were also carried out with the protein in the presence of a number of different ligands including coumarate and ferulate. There was clear electron density in the maps for the coumarate and ferulate ligands. The coumarate-bound structure was determined to 2.5 Å resolution and is identical to a 1.6 Å structure of CouP with coumarate recently deposited in the PDB (entry 4F8J), apart from minor changes at the termini. Thus, we will not discuss its structure further here, other than to note that the mode of ligand binding is identical to that for ferulate. A summary of the relevant data processing and refinement statistics for the ferulate complex and the ligand free form are presented in Table 3, and the 1.9 Å structure of CouP with ferulate bound is shown in Figure 5. The structure displays all the features expected of an ABC-bacterial periplasmic binding protein; namely a two-domain structure in which an intrinsic cleft positioned between the two domains forms the ligand-binding pocket (Fig. 5a).

**Table 3:** Crystallographic data and refinement parameters.

	Ligand free	Ferulate bound	
**Data collection**			
Space group	P 2_1_2_1_2_1_	P 2_1_2_1_2_1_	
Unit Cell (Å)	a = 42.9 b = 66.4 c = 100.5	a = 43.0 b = 70.7 c = 105.3	
Resolution range (Å)	43.0–2.1(2.15–2.10)	44.3–1.9(1.96–1.91)	
No of measured reflections	61078 (4579)	90559 (6819)	
No of unique reflections	31821 (2389)	46986 (3551)	
Completeness (%)	98.0 (99.3)	97.5 (98.9)	
R­_meas_ * (%)	10.5 (66.4)	9.9 (65.2)	
Mn<I/sd>	8.1 (1.9)	8.8 (1.6)	
**Refinement**			
R/R_free_^ 2,3^	0.17/0.23	0.18/0.23	
Overall B-factor (Å^2^)	29.4	20.3	
RMSD in bond distances (Å)	0.019	0.021	
RMSD in bond angles (°)	1.8	1.7	
**Ramachandran**			
% most favored	96	97	
% additionally allowed	4	3	

* Rmeas is a multiplicity weighted R-factor measure of the agreement of symmetry related reflections as reported by XSCALE.

**Figure 6:**
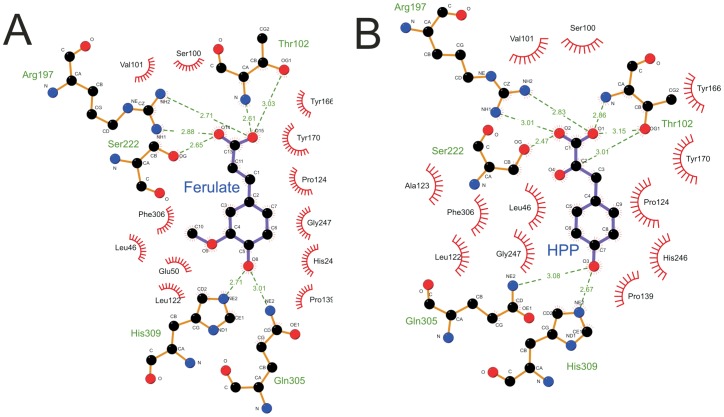
Ligplot representations of the interactions of (A) ferulate and (B) 4-hydroxyphenylpyruvate with CouP (RPA1789). The key interactions are the H-bonds formed by His309 and Gln305 to the 4-OH group on the aromatic ring and also the H-bonds formed by Arg197/Thr102/Ser222 to the oxygen atoms of the carboxyl group of the ligand side-chain.

Analysis of the interactions in the binding cleft show that the aromatic ring of the ligand is bound most deeply. Key H-bond interactions are formed between the 4-OH group of the aromatic ring of ferulate with His309 and Gln305 residues (Fig. 5b and Fig. 6). H-bond interactions are also made between the carboxyl group on the ferulate side chain and Arg197, Ser222 and Thr102. These latter interactions explain why compounds such as 4-hydroxybenzoate showed no evidence of binding; they lack the 3-carbon chain that ensures optimal bond distances on each side of the binding cleft are formed.

Cinnamate had the weakest binding affinity to CouP of the four major aromatic ligands used in this study, and the structural data show that as cinnamate does not possess a functional group on its aromatic ring it would not be able to form the H-bond interactions with His309 and Gln305 that the other compounds can, which is reflected in its higher *K*_d_ value (Table 1) and less favourable enthalpy of binding (Table 2). The methoxy-group possessed by ferulate sits in a pocket bounded by the side chains of Leu46, Phe306 and His309 and the main chain carbonyl oxygen atoms of Ser100 and Leu122. There are also close ligand contacts with Glu50, Phe306 and His309 (Fig 5b and Fig. 6).

These structural data satisfactorily explain the relative binding affinities of the lignin-derived aromatic monomers, whereby the functional groups they possess (particularly the 4-OH group) on their aromatic rings dictate strength of binding. The data also explain how structurally similar ligand contaminants like 4-HPP can be incorporated into the binding pocket.

### Fluorescence Spectroscopy Shows that RPA1782 Binds the Same Range of Aromatic Ligands as CouP

**Figure 7:**
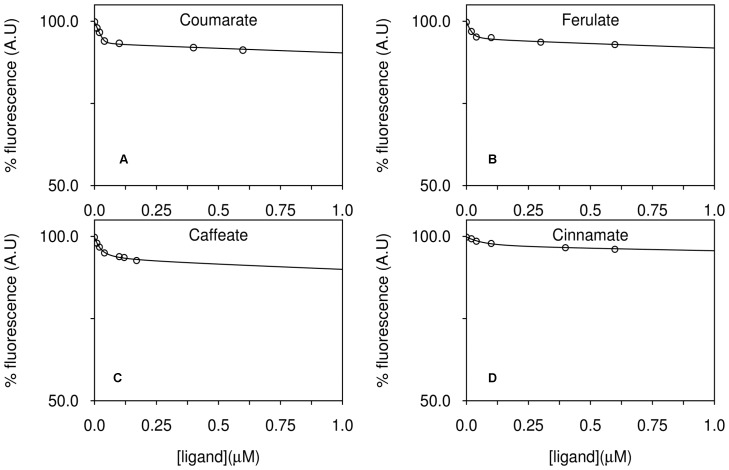
Fluorescence titrations of TarP (RPA1782) with lignin-derived aromatic ligands. The fluorescence emission of 0.2 µM TarP was followed at 340 nm during titrations with (A) coumarate, (B) ferulate, (C) caffeate and (D) cinnamate. In each case, data were fit to a single-site binding model and dissociation curves were plotted. The figure shows individual representative titrations.

The location of the *rpa1782-1784* genes in the same locus as the catabolic genes for the non-β oxidation pathway led to an investigation of the role of this TRAP transporter in the uptake of aromatic compounds. RPA1782 was much harder to overproduce in a soluble form compared to CouP, and although expression in the pBAD system was successful, it gave low yields of protein contaminated with GroEL. This was successfully removed as described in Materials and Methods. Purified recombinant RPA1782 was initially screened for potential ligands in the same manner as CouP and evidence was obtained that coumarate, caffeate, ferulate and cinnamate could all bind to the protein, as shown by a ∼5% quench in fluorescence with equimolar protein and each ligand in each case; Fig. 2b shows a typical example of the coumarate-induced quench. Like CouP, 4-hydroxybenzoate showed no evidence of binding. Ligand titrations were performed with coumarate, caffeate, ferulate and cinnamate by monitoring fluorescence emission at 340 nm (Fig. 7 and Table 1). Due to the much smaller fluorescence quench compared to CouP, these titrations had larger errors, but from the set of *K*_d_ values calculated for RPA1782 the data clearly indicate sub-micromolar binding affinities for each ligand (Table 1). These results are strikingly similar to those gained for CouP; the relative *K*_d_ values for each ligand are of the same order and follow the same pattern for both proteins. Thus, RPA1782 shows the tightest binding for coumarate, caffeate and ferulate, with cinnamate displaying the weakest binding (Table 1). Like CouP, RPA1782 was also found to bind 4-HPP, with a *K*_d_ of 42±7 nM. Problems with the yield, solubility and stability of recombinant RPA1782 protein meant that further analysis by ITC was precluded. However, it is clear that this protein also functions in the binding of aromatic compounds as part of a TRAP transporter, so following the original nomenclature introduced by Forward *et al*. [15], we propose to designate the components of this transporter TarP (TRAP transporter for aromatic compounds, periplasmic protein, RPA1782), TarQ (small transmembrane transport protein, RPA1783) and TarM (large transmembrane transport protein, RPA1784).

## Discussion

In this study we have demonstrated that the periplasmic binding-proteins from two genetically and structurally distinct transport systems in *R. palustris* have identical substrate specificity for key lignin-derived aromatic compounds that this bacterium can use as growth substrates. Most strikingly, these proteins display remarkably similar *K*_d_ values for the substituted cinnamic acids studied here, all of which were in the nanomolar range as measured by fluorescence spectroscopy (and additionally ITC in the case of CouP). It is important to note that while CouP is homologous with proteins in the LivK family of ABC-type periplasmic binding-proteins, TarP is homologous with the DctP family of periplasmic binding-proteins [15]. While their overall fold is likely to be similar, they possess insignificant sequence similarity, which indicates that such high affinity binding of the same ligands has been achieved by the evolution of different binding mechanisms. Evidence for this in relation to TarP is discussed below.

The measured *K*_d_ values for CouP were well determined and obtained in experiments with protein:ligand stoichiometries approaching unity. However, it was important to remove any endogenously bound ligand by unfolding and refolding the protein, as it was clear from our initial crystallographic data that 4-HPP can occupy the binding cleft. This ligand is a metabolic intermediate in tyrosine metabolism [29] and we assume that it becomes incorporated during recombinant protein production in the *E. coli* cytoplasm. In this context, the binding data obtained with CouP and coumarate was especially interesting, as a recent study on this protein reported a coumarate *K*_d_ value of 8.6 µM as measured by ITC [18]. In our study, we independently confirmed, using both fluorescence and ITC methods that CouP has a much higher affinity for this key ligand.

With both proteins the highest affinities were observed for coumarate, caffeate and ferulate, while cinnamate itself had a rather lower affinity. These data indicate that the possession of a 4-OH group on the aromatic ring considerably enhances ligand binding, which is reinforced by the structural evidence gained from X-ray crystallographic studies with CouP. This revealed interactions of the 4-OH group on the aromatic ring of ferulate with His309 and Gln305 residues in the binding cleft. As expected, these hydrogen bonds contribute favourably to the enthalpy of binding, resulting in the considerably less favourable enthalpy of binding for cinnamate (ΔH_bind_>4 kcal mol^−1^), which lacks the 4-OH moiety, thus resulting in a much weaker affinity with CouP. The introduction of a second hydroxyl, as in caffeate, further decreases the enthalpy of binding, such that at 20°C there is no net contribution from changes in bonding, strongly suggesting that further hydrogen bonds are made to the 3-OH moiety (as supported by a crystal structure of this complex recently deposited in the PDB, code 4FB4). Caffeate also has the least favourable entropy of binding of the four ligands (Table 2), indicating that the third H-binding moiety considerably increases the order of the system, possibly related to the ordering of a water molecule to form the H-bond to the 3-OH. Altering this group to a methoxy, as in ferulate, removes its H-bonding potential and thus the favourable enthalpy component, to give the same ΔH of binding as coumarate. The entropy of binding is not altered, resulting in weaker dissociation constant for ferulate than either coumarate (which has a more favourable ΔS) or caffeate (which has more favourable ΔH). In conclusion, electrostatic contributions from ring substituents contribute substantially to the binding energy, above a fairly constant favourable entropic contribution, driven by the hydrophobic effect. It should be noted that this thermodynamic analysis refers only to single temperature measurements and conclusions may be significantly altered if the heat capacities of binding for the ligands vary significantly.

**Figure 8:**
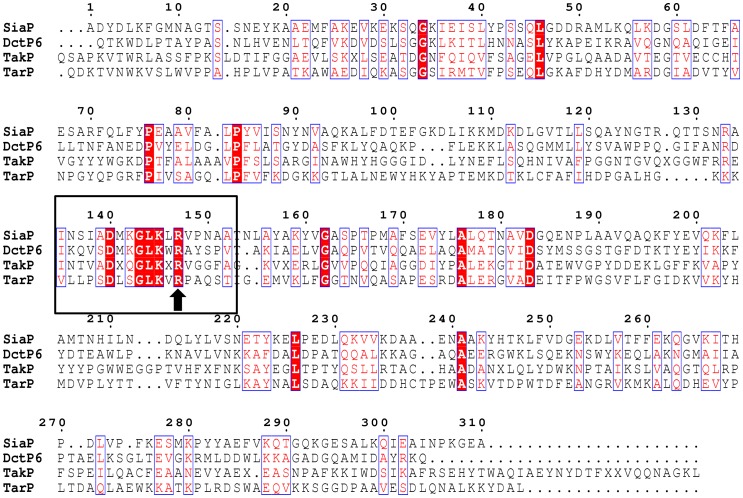
Multiple sequence alignment of TarP with selected homologues with solved crystal structures. The alignment was generated with CLUSTAL W; identical residues are highlighted white in red boxes, similar residues are highlighted red. A conserved arginine that was first shown [32], [33] to form a salt bridge with the carboxylate of sialic acid bound to SiaP (Arg147) is indicated with an arrow and a region of sequence conservation surrounding this residue is indicated within the boxed area. This arginine residue is thought to be crucial in all TRAP transporter binding-proteins that interact with a ligand cotaining a carboxylate group. The proteins are: SiaP – *Haemophilus influenzae* (sialic acid); DctP6– *Bordetella pertussis* (pyroglutamate); TakP – *Rhodobacter sphaeroides* (pyruvate); TarP- *Rhodopseudomonas palustris* (cinnamic acids). The N-terminal signal sequences have been removed for this alignment (predicted with the SignalP server) and the residue numbering shown corresponds to that of SiaP. The conserved arginine in the mature TarP is Arg149, corresponding to Arg168 in the pre-protein.

Interestingly, although the ABC-type and TRAP transport systems studied here both use a periplasmic binding protein as the initial solute receptor, they otherwise represent structurally and mechanistically different solutions to the problem of the uptake of aromatic compounds from the environment. While ABC systems are primary systems powered by ATP hydrolysis, TRAP systems are secondary transporters energised by either a proton or sodium gradient across the cytoplasmic membrane [15], [30], [31]. The first TRAP transporter to be identified and characterised was the Dct system in the related purple photosynthetic bacterium *Rhodobacter capsulatus*, for the uptake of the C4-dicarboxylates malate, succinate and fumarate [15]. Subsequently, TRAP transporters have been found to be extremely widespread in many groups of bacteria and archaea [30] but a common characteristic that is exemplified by the *R. palustris* system described here, is their transport of carboxylic acid substrates [30], [32]. From structural studies and sequence alignments of members of the DctP-type binding protein family it has become clear that the formation of a salt bridge between a highly conserved arginine residue in the binding pocket and the carboxylate group of the ligand is crucial to defining this substrate preference [32]. Several determined structures of DctP-type binding proteins with bound ligand clearly reveal this salt bridge [33]–[35]. For example, SiaP from *Haemophilus influenzae* forms a salt bridge with its favoured ligand sialic acid at Arg147 [33] and a sequence alignment (Figure 8) between SiaP,TarP and other homologues highlights Arg149 in TarP as the conserved residue (mature protein numbering). Although no phenylpropeneoid aromatic compounds have yet been visualised in the binding pocket of a DctP-type binding protein, the cinnamic acid derivatives used as ligands in this study all possess a carboxylate group positioned on the alpha carbon of their side chain so would have the ability to form a salt bridge with this conserved arginine residue. We therefore propose that these compounds would be bound in the TarP binding pocket with the carbon chain buried most deeply; this would be in opposition to CouP, which buries the aromatic ring most deeply in the binding pocket (Fig. 5). Only structural studies of TarP will confirm this; so far we have been unsuccessful in obtaining diffracting crystals of ligand-bound protein.

Our results with these two binding-proteins are consistent with evidence that both the genes for the TRAP and ABC transport systems are inducible by *p*-coumarate [13] and are members of the same regulon, controlled by CouR, along with the genes encoding the enzymes of the non β-oxidation pathway with which they are clustered on the *R. palustris* chromosome [10], [16]. Such regulation underlines the intimate coupling of transport of aromatic substrates into the cell with their metabolism. These phenylpropeneoid monomers are clearly too polar to diffuse through the cytoplasmic membrane and the existence of aromatic transport systems indicates that *R. palustris* needs active import of such metabolites in order to achieve competitive growth rates on such compounds in its natural environment. The nanomolar affinities of the binding proteins of these systems would, moreover, allow the bacterium to scavenge very low environmental concentrations of a range of substituted cinnamic acids derived from lignin. It is not obvious, however, why *R. palustris* employs two such systems with an essentially identical substrate range and similar regulation of expression. The answer to this transporter redundancy may lie in their different energy coupling mechanisms, so that aromatic substrate uptake is still guaranteed, for example, in times of fluctuating ATP availability or transiently low membrane potential.
